# Splenic Volume, an Easy-To-Use Predictor of HCC Late Recurrence for HCC Patients After Hepatectomy

**DOI:** 10.3389/fonc.2022.876668

**Published:** 2022-05-24

**Authors:** Tongdi Fang, Guo Long, Xingyu Mi, Wenxin Su, Lei Mo, Ledu Zhou

**Affiliations:** ^1^ Department of General Surgery, The Xiangya Hospital of Central South University, Changsha, China; ^2^ National Clinical Research Center for Geriatric Disorders, Xiangya Hospital, Central South University, Changsha, China

**Keywords:** splenic volume (SV), hepatocellular carcinoma, recurrence, nomogram, liver cirrhosis

## Abstract

**Purpose:**

The high recurrence rate of hepatocellular carcinoma (HCC) has a poor impact on the quality of life and survival time of patients. Especially for late recurrence, poor data are available in analysis. We aim to evaluate whether the splenic volume (SV) measured from preoperative CT images could predict late recurrence in HCC patients after hepatectomy.

**Patients and Methods:**

A cohort of 300 HCC patients hospitalized at Xiangya Hospital of Central South University between January 2015 and June 2018 was retrospectively analyzed. The SV was calculated by using automated volumetry software from preoperative CT images. A total of 300 HCC patients were separated into the early recurrence cohort (n=167), the late recurrence cohort (n=39), and the no recurrence cohort (n=94) according to whether there is a recurrence and the recurrence time. Univariate and multivariate Cox analyses were performed to identify the independent risk factors of both early and late recurrence.

**Results:**

AFP, Microvascular invasion (MVI), satellitosis, and BCLC staging were independent risk factors of HCC early recurrence. Splenic volume (HR=1.003, 95%CI:1.001-1.005, P<0.001) was the only predictor of HCC late recurrence. Based on X-tile software, 133 non-early recurrence patients were divided into two groups according to SV: low SV (<165ml, n=45) and high SV (≥165ml, n= 88). The low SV group had a significantly better RFS compared with the high SV group (P=0.015). Nomogram was built on the base of SV to get the probability of 3-year RFS, 4-year RFS, and 5-year RFS.

**Conclusion:**

In our study, we drew a conclusion that splenic volume was the only predictor of HCC late recurrence because of its association with portal hypertension and liver cirrhosis. High splenic volume often indicated a worse recurrence.

## Introduction

In the past few decades, hepatocellular carcinoma (HCC) has risen to become the sixth most aggressive malignancy and the fourth-leading cause of cancer-related deaths worldwide (1). Among the various therapeutic approaches, hepatic resection (HR) is presently considered one of the most curative modalities for HCC patients (2). With the continuous improvement of perioperative management and operation techniques over the past few years, although the overall survival time of the patient has been prolonged, the recurrence of HCC after hepatectomy remains high (3). As reported by the literature, the cumulative 5-year recurrence rate for HCC patients after hepatic resection was approximately 80% in most centers (4, 5).

The potential mechanism of postoperative recurrence could be an intrahepatic relapse of the primary tumor (considered as early recurrence) or the emergence of *de novo* multicentric tumor (stated as late recurrence). Therefore, according to the time to postoperative recurrence, we artificially divided the recurrence of liver cancer into early recurrence (recurrence within 24 months) and late recurrence (recurrence over 24 months) (6–9). Numerous recent studies have shown that early recurrence is related to biological characteristics of tumors such as tumor size, tumor numbers, tumor differentiation, microscopic vascular invasion (MVI), and satellitosis (10–12). There is a lack of data to predict late recurrence. Scholars tend to accept the view that late recurrence is commonly associated with the intrinsic liver condition, like liver cirrhosis (13, 14).

Liver cirrhosis is closely related to portal hypertension (PH) and PH could influence the natural history of the underlying liver condition in return (15). We couldn’t accurately assess PH clinically without some invasive methods. The spleen is a sensitive organ to PH alteration and responds promptly in the form of splenomegaly. Moreover, splenomegaly can contribute to the progression of PH by increasing splenic blood flow (16). However, the relationship between splenomegaly and liver cirrhosis or PH is controversial. This is partly because the spleen is irregular in shape and the spleen size is measured by longitudinal spleen axis on ultrasound or CT images, which makes it not as accurate as three-dimensional measurement.

Recently, liver computed tomography (CT) volumetry has been used to help assess liver volume in clinical practice (17, 18). Especially, when considering the future liver remnant (FLR) volume, we can use three-dimensional CT reconstruction to accurately represent the remaining liver volume (19). Similarly, with the same use of three-dimensional CT volumetry software, the splenic volume could be feasible to obtain. Some studies have shown that spleen volume measured by CT is a dependable indicator for liver fibrosis, cirrhosis, and portal hypertension (20). As far as we know, the role of splenic volume as a predictor of HCC late recurrence has never been explored. Therefore, this study aimed to assess whether the SV measured by 3D reconstruction could predict HCC late recurrence after hepatectomy.

## Patients and Methods

### Patients

A total of 320 patients meeting the inclusion criteria had undergone curative liver resection between January 2015 and June 2018 at XiangYa Hospital. Considering the exclusion criteria, we excluded 11 patients due to recurrent tumors and nine patients were excluded because of receiving anticancer treatments prior to hepatectomy. None of the patients had splenomegaly due to other causes such as hematological diseases. At last, our study included 300 HCC patients who underwent curative liver resection between January 2015 and June 2018 at XiangYa Hospital, Central South University.

Before hepatectomy, the splenic volume was measured using three-dimensional CT reconstruction for each patient. The study was under the permission of the Ethics Committee of the Xiangya Hospital and complied with the Declaration of Helsinki. Because of the retrospective nature of the study, the ethics committee did not require patient consent to review their medical data. The selection of HCC patients for hepatectomy in our study was based on Barcelona Clinic Liver Cancer (BCLC) criteria.

The inclusion criteria included: 1) diagnosis of HCC; 2) no simultaneous malignancies; 3) R0 resection confirmed by the postoperative pathological examination; 4) patients underwent a CT examination before surgery; 5) aged above 18 years old.

The exclusion criteria included: 1) patients were considered to have recurrent tumors; 2) patient received radiofrequency ablation (RA), transarterial chemoembolization (TACE), or other anticancer treatments before hepatectomy; 3) splenomegaly from other causes, like hematological diseases.

### Follow-up and Endpoints

Routine examinations were required for all patients every 2 to 3 months after surgery in the first 2 years, including liver function tests, tumor markers, and liver ultrasound. A confirmatory computed tomography (CT) and/or magnetic resonance imaging (MRI) scan were performed if necessary. After 2 years of follow-up, we reduced the frequency of follow-up visits to every 3 to 6 months. The endpoint of the study was recurrence-free survival (RFS), which was measured as the time from the end of surgery to recurrence or metastasis (including death due to recurrence or metastasis), or the last follow-up date. The patients with a recurrence or metastasis within 24 months after hepatectomy were allocated into the early recurrence cohort, and those with a relapse or metastasis over 24 months were put into the late recurrence cohort. The patients without relapse or metastasis until the last follow-up date were allocated into the no recurrence cohort. Patients followed up for 2 years at least or having recurrence after surgery were collected. The follow-up endpoint was July 1, 2020.

### Splenic Volume Calculation

**Figure 1:**
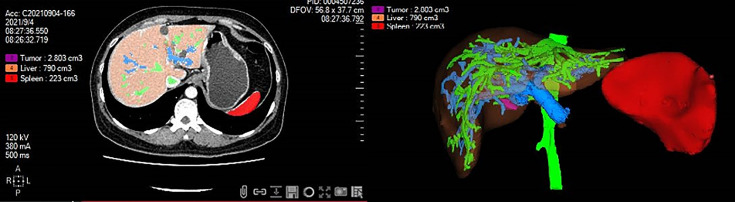
Three-dimensional reconstruction of the spleen was performed for automated volumetry by using medical image analysis software. The splenic volume was calculated after volumetry of the spleen. Red color, spleen; Purple color, tumor; Orange color, liver; Green color, hepatic vein; Blue color, portal vein.

All patients received an abdominal CT scan before surgery. By using a medical image analysis procedure (Myrian^®^; Intrasense S.A.S., Montpellier, France), a three-dimensional reconstruction of the spleen was performed for automated volumetry, and the results were modified by manual tracking of splenic margin following automated reconstruction by two experienced observers. The final SV was calculated after volumetry of the spleen (example shown in **Figure 1**).

### Clinicopathologic Variables

The following factors were analyzed: age (time of surgery), gender, preoperative serum examination including routine blood, liver function, coagulation function, serum alpha-fetoprotein (AFP), hepatitis B surface antigen (HBsAg), spenic volume, and postoperative clinicopathological data including tumor size, tumor number, tumor differentiation, satellitosis, liver cirrhosis, microvascular invasion (MVI), and tumor stage (BCLC staging).

### Statistical Analysis

We collected patients who were followed up for 2 years to identify the independent factors associated with early recurrence. Then, those without relapse within 2 years were reanalyzed to recognize the independent variables related to late recurrence.

Continuous variables were expressed as mean ± standard deviation and analyzed using t-tests. Categorical variables were shown as frequency (percentage) and chi-square test was performed for comparison. Univariable and multivariate Cox analyses were performed for identifying the associated factors of RFS. In order to verify the significance of our findings and their potential application in clinical practice, we built a prediction model (nomogram) based on the independent late recurrence predictors by using the *R* (version 4.0.3). According to optimal cutoff values determined by X-tile software, the included patients were divided into two sub-groups. Recurrence-free survival (RFS) analyses were assessed by using the Kaplan-Meier (K-M) curve and a log-rank test. Statistical analysis was performed using SPSS 26.0 for windows (SPSS, Chicago, IL, USA) and R 4.1.1 software (Institute for Statistics and Mathematics). P< 0.05 was considered statistically significant.

## Result

### Clinicopathologic Characteristics

**Figure 2:**
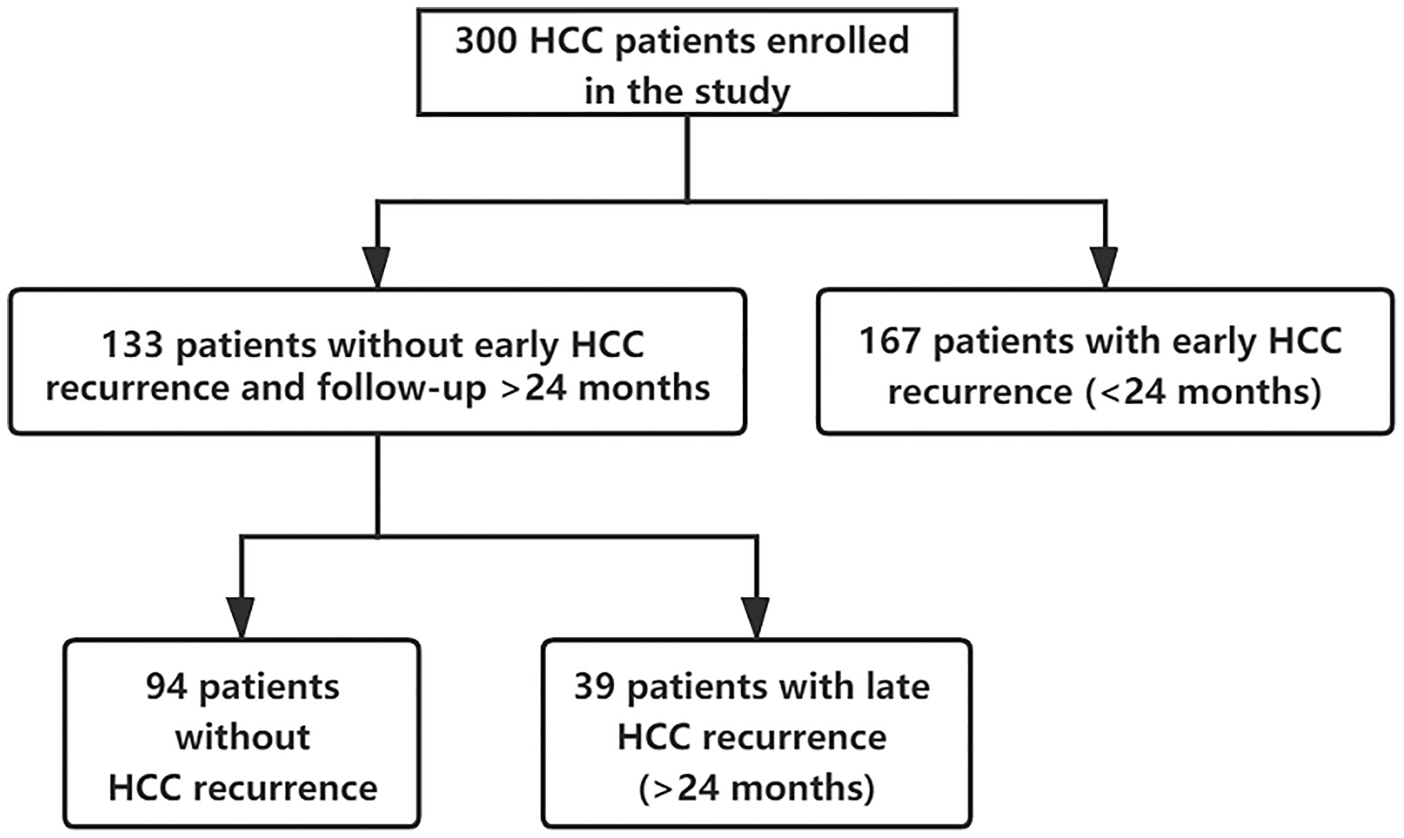
Flowchart of this study.

A total of 300 patients who met the inclusion and exclusion criteria were included. They were followed up for at least 2 years from the surgery date until the last follow-up date or until having HCC recurrence. Clinicopathologic characteristics of the three groups are comparatively shown in **Table 1**. Of 300 included patients, 167 out of 300 (55.7%) patients had an early recurrence, 39 out of 300 (13.0%) patients developed a late recurrence, and 94 (31.3%) patients didn’t have recurrence (shown in **Figure 2**).

**Table 1:** Demographics and clinical data of study population.

	Whole cohort (N=300)	Early recurrence (N=167)	Late recurrence (N=39)	No recurrence (N=94)
Age, yr (mean ± SD)	50.38 ± 11.02	48.96 ± 11.54	52.40 ± 8.84	52.14 ± 10.58
Male, n (%)	261 (87)	146 (87.4)	36 (92.3)	79 (84)
Female, n (%)	39 (13)	21 (12.6)	3 (7.7)	15 (16)
HBsAg (+), n (%)	255 (85)	149 (89.2)	32 (82.1)	74 (78.7)
Cirrhosis, n (%)	227 (75.7)	133 (79.6)	31 (79.5)	63 (67)
Neutrophil, 10^9^L (mean ± SD)	3.47 ± 1.34	3.53 ± 1.40	3.12 ± 1.37	3.48 ± 1.21
Lymphocyte,10^9^L (mean ± SD)	1.52 ± 0.67	1.52 ± 0.79	1.39 ± 0.41	1.58 ± 0.49
Platelet,10^3^L (mean±SD)	167.00 ± 70.94	172.50 ± 78.52	140.53 ± 67.27	168.10 ± 54.16
TBil, μmol/L (mean ±SD)	13.13 ± 6.02	13.58 ± 7.05	12.27 ± 4.21	12.62 ± 4.46
Alb, g/L (mean ±SD)	41.26 ± 4.59	40.79 ± 4.86	41.93 ± 4.72	41.75 ± 3.99
ALT, U/L (mean ±SD)	39.91 ± 26.09	41.50 ± 26.58	41.27 ± 18.95	36.40 ± 27.56
AST, U/L (mean ±SD)	47.44 ± 34.47	52.78 ± 38.36	41.90 ± 20.35	40.09 ± 30.07
PT, s (mean ±SD)	13.55 ± 1.10	13.57 ± 1.13	13.68 ± 0.97	13.46 ± 1.10
Tumor size, cm (mean ±SD)	6.38 ± 3.97	7.42 ± 4.11	5.29 ± 3.14	4.97 ± 3.47
AFP, ng/ml, n (%) ≤400 >400	191 (63.7) 109 (36.3)	91 (54.5) 76 (45.5)	30 (76.9) 9 (23.1)	70 (74.5) 24 (25.5)
Tumor number, n (%) 1 >2	198 (66.0) 102 (34.0)	89 (53.3) 78 (46.7)	30 (76.9) 9 (23.1)	79 (84) 15 (16)
MVI, n (%) No Yes	169 (56.3) 131 (43.7)	77 (46.1) 90 (53.9)	27 (69.2) 12 (30.8)	65 (69.1) 29 (30.9)
Satellitosis, n (%)	65 (21.7)	55 (32.9)	4 (10.3)	6 (6.4)
Splenic volume, mL (mean ±SD)	260.22 ± 160.89	272.47 ± 174.92	304.50 ± 178.83	219.19 ± 111.36
BCLC staging, n (%) A B C	187 (62.3) 67 (22.3) 46 (15.3)	76 (45.5) 51 (30.5) 40 (24)	29 (74.4) 7 (17.9) 3 (7.7)	82. (87.2) 9 (9.6) 3 (3.2)
Differentiation, n (%) Well Moderate Poor	13 (4.3) 226 (75.3) 61 (20.3)	3 (1.8) 123 (73.7) 41 (24.6)	1 (2.6) 34 (87.2) 4 (10.3)	9 (9.6) 69 (73.4) 16 (17)
Recurrence, n (%)	206 (68.7)	167 (55.7)	39 (13.0)	0

AFP, alpha-fetoprotein; ALB, albumin; ALT, alanine transaminase; TBIL, total bilirubin; AST, aspartate transaminase; MVI, microvascular invasion; PT, prothrombin time; BCLC, Barcelona Clinic Liver Cancer; HBsAg, hepatitis be antigen.

In the whole cohort, the average age at diagnosis was 50.4 ± 11.0 years old. The majority of patients (87%) were male and 85% of patients were infected with HBV, and cirrhosis was observed in 227 (75.7%) patients. The mean size of the tumor was (6.38 ± 3.97) cm and 102 (34.0%) patients had multiple tumors. The mean splenic volume was (260.22 ± 160.89) ml. Between three sub-group cohorts, the early recurrence cohort had a larger tumor size (7.42 ± 4.11) cm and a higher proportion of MVI (53.9%), satellitosis (32,9%), multiple tumors (46.7%), and poor tumor differentiation (24.6%). The late recurrence cohort had a larger splenic volume (304.50 ± 178.83) ml than others.

As for short-term complications, 75 Complications occurred in 50 patients among 300 HCC patients. Among them, liver failure was observed in 30 (10%) patients, with pleural effusion in 15 (5%) patients, biliary fistula in 11 (3.6%) patients, pulmonary infection in 10 (3.3%) patients, abdominal bleeding in six (2%) patients, and upper gastrointestinal bleeding in three (1%)patients.

### Analyses of Risk Factors for HCC Recurrence

#### Early Recurrence (<2 Years)

**Figure 3:**
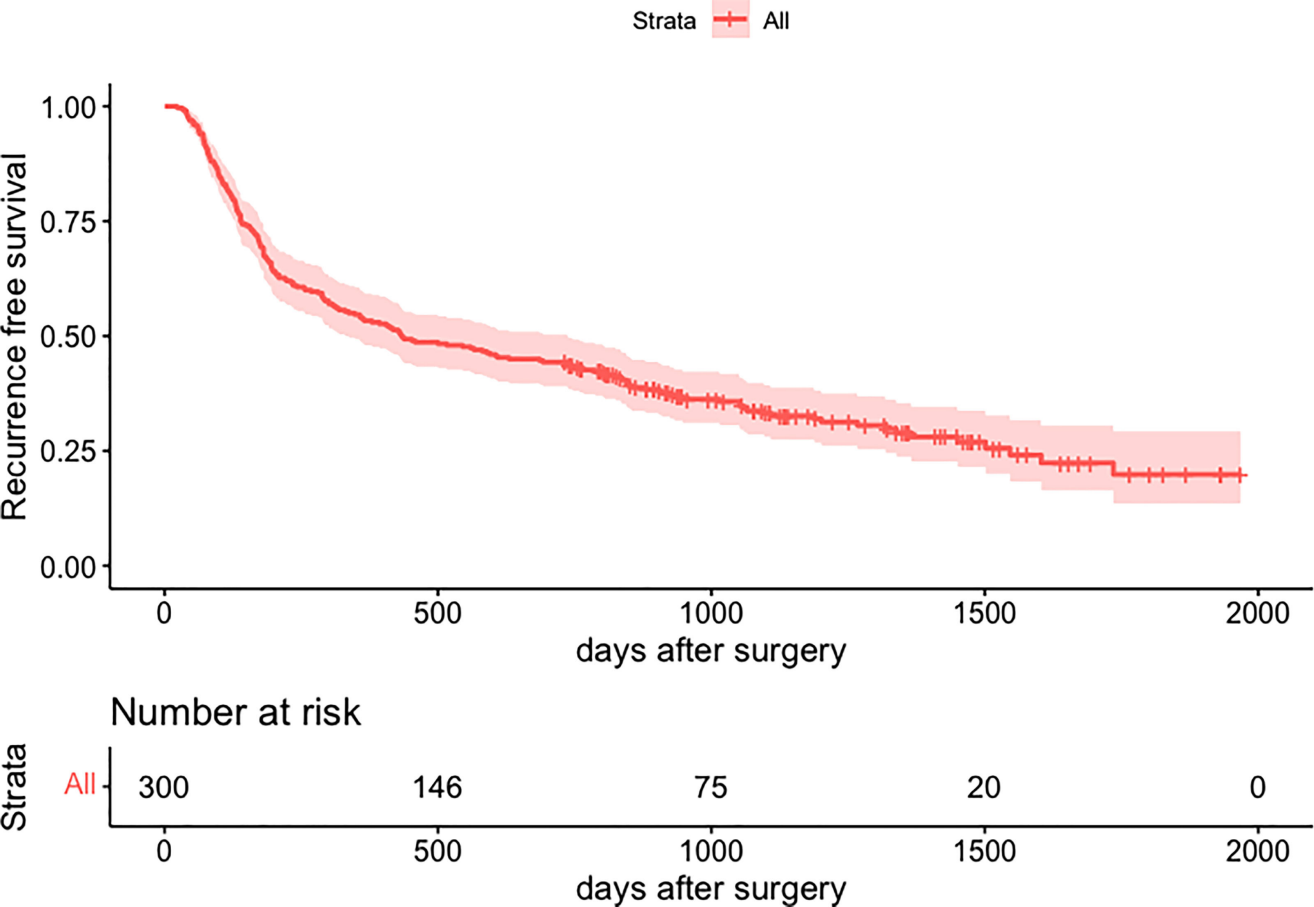
Kaplan-Meier curve for recurrence-free survival of 300 patients. HCC, hepatocellular carcinoma.

Total clinicopathologic factors of 300 patients were taken into univariate and multivariate Cox analyses to identify independent risk factors. The univariate Cox analysis revealed that age, HBsAg, platelet, TBil, AST, AFP, tumor size, tumor number, MVI, satellitosis, BCLC staging, and tumor differentiation were potential risk factors of HCC early recurrence. Then, all the above factors were taken into multivariate Cox analysis. It suggested that AFP, MVI, satellitosis, and BCLC staging were independent risk factors of HCC early recurrence (shown in **Table 2**). The curve of RFS is shown in **Figure 3**.

**Table 2:** Univariable and multivariable analyses for independent variables associated with HCC early recurrence.

Variables	Univariable cox regression		Multivariable cox regression	
N=300	HR (95%CI)	P value	HR (95%CI)	P value
Age, years	0.979 (0.965-0.993)	0.004	0.995 (0.979-1.011)	0.311
Gender, (Female vs Male)	0.987 (0.625-1.559)	0.955		
HBsAg, (Yes vs No)	1.656 (1.015-2.702)	0.043	1.425 (0.849-2.393)	0.144
Cirrhosis, (Yes vs No)	1.380 (0.947-2.012)	0.094		
Neutrophil, 10^9^/L	1.068 (0.957-1.191)	0.241		
Lymphocyte, 10^9^/L	0.976 (0.765-1.244)	0.842		
Platelet, 10^9^/L	1.003 (1.001-1.005)	0.013	1.002 (1.000-1.005)	0.127
TBil, μmol/L	1.027 (1.003-1.052)	0.029	1.018 (0.995-1.041)	0.196
Alb, g/L	0.967 (0.934-1.001)	0.057		
ALT, U/L	1.003 (0.998-1.008)	0.292		
AST, U/L	1.006 (1.003-1.010)	<0.001	0.999 (0.993-1.004)	0.915
PT, s	1.031 (0.893-1.189)	0.679		
AFP, (>400 vs ≤ 400ng/ml)	2.143 (1.578-2.910)	<0.001	1.758 (1.286-2.404)	**<0.001**
Tumor size, cm	1.094 (1.061-1.128)	<0.001	0.998 (0.950-1.049)	0.811
Tumor number, (≥2 vs 1)	2.452 (1.805-3.332)	<0.001	1.332 (0.714-2.482)	0.566
MVI, (Yes vs No)	2.138 (1.576-2.902)	<0.001	1.423 (1.020-1.985)	**0.038**
Satellitosis	2.795 (2.017-3.875)	<0.001	1.551 (1.031-2.333)	**0.035**
Splenic volume (mL)	1.000 (1.000-1.001)	0.280		
BCLC staging		<0.001		**<0.001**
A	reference		reference	
B	2.795 (1.954-3.998)		1.920 (1.229-2.999)	
C	4.512 (3.058-6.659)		3.114 (2.007-4.833)	
Differentiation		0.006		0.244
well	reference		reference	
moderate	3.127 (0.994-9.832)		2.134 (0.673-6.771)	
poor	4.793 (1.483-15.489)		2.457 (0.741-8.149)	

AFP, alpha-fetoprotein; HBsAg, hepatitis be antigen; ALB, albumin; ALT, alanine transaminase; TBIL, total bilirubin; AST, aspartate transaminase; PT, prothrombin time; MVI, microvascular invasion; BCLC, Barcelona Clinic Liver Cancer; HR, Hazard Ratio. ‘P < 0.05’ was highlighted in bold.

#### Late Recurrence (>2 Years)

**Figure 4:**
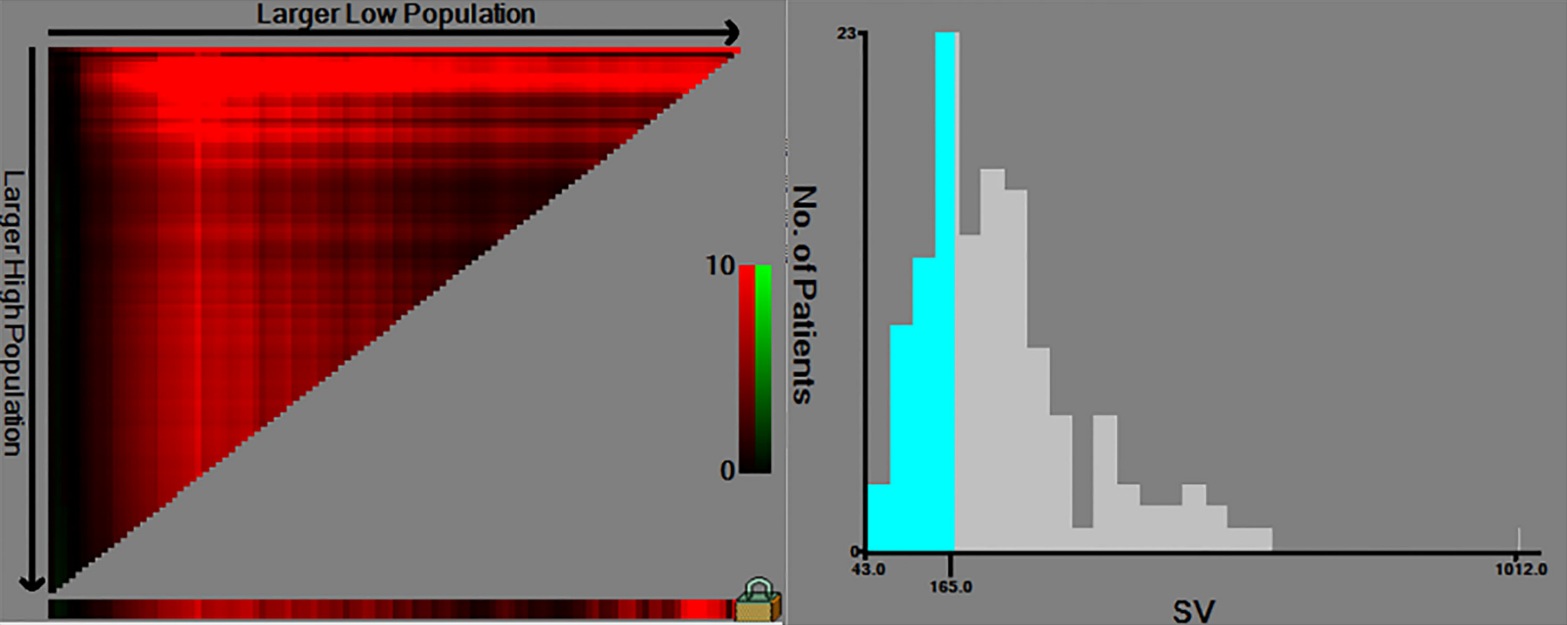
The cut-off values were calculated by using X-tile based on the SV. The cut-off values were 167 for RFS. SV, splenic volume; RFS, recurrence-free survival.

**Figure 5:**
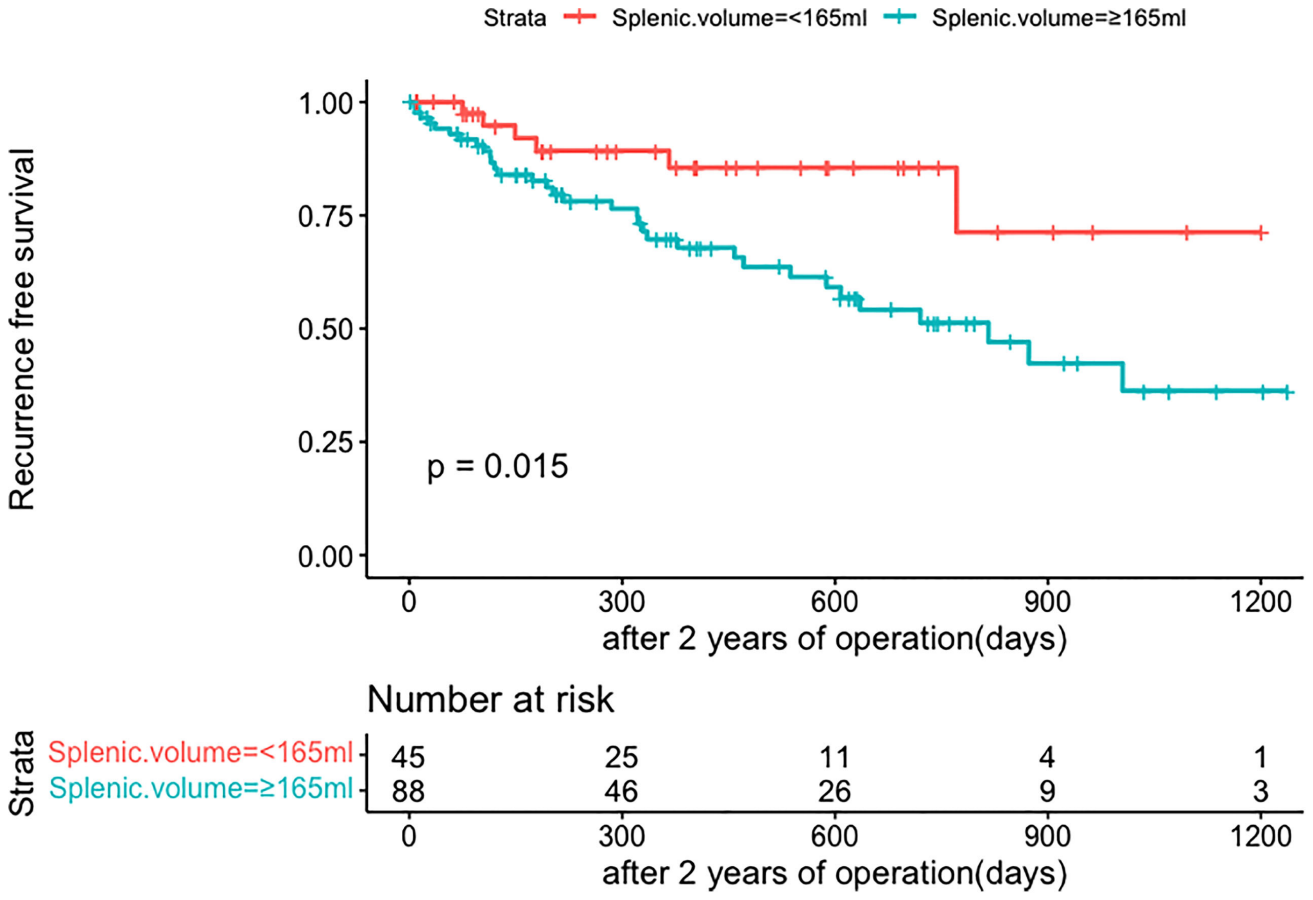
Comparison of RFS in late recurrence patients between two groups (SV<165ml, SV≥165ml). RFS, recurrence-free survival; SV, splenic volume.

The same clinicopathologic factors of 133 patients were taken into univariate and multivariate Cox analyses to identify independent risk factors. The univariate analysis suggested that platelet (P=0.038) and splenic volume (P=0.001) were potential risk factors of HCC late recurrence. For further multivariate analysis, only splenic volume (HR=1.003, 95%CI:1.001-1.005, P=0.001) was an independent risk factor of HCC late recurrence (shown in **Table 3**). (HR=1.003 means: for every 1 mL increase in splenic volume, a 0.3% increase in risk of late recurrence (0.3% as 1.003-1 = 0.003)). With the help of X-tile software, we figured splenic volume = 165 ml as an optimal cut-off value. Based on this cut-off value, the 133 patients were divided into two groups: low SV (<165ml, n=45), and high SV (≥165ml, n= 88) (shown in **Figure 4**). Subsequently, the survival analysis of RFS was performed by Kaplan–Meier method between two groups (shown in **Figure 5**). Among patients who survived without a recurrence within 24 months (time origin from 24 months), the median RFS was 816 days (95% CI: 544–1087 days) in the high SV group and it didn’t reach the median RFS in the low SV group. The low SV group had a significantly better RFS compared with the high SV group (P=0.015). Especially, 3- and 5-year RFS rates were 70% and 36% in the high SV group, while they were up to 89% and 71% in the low group.

**Table 3:** Univariable and multivariable analyses for independent variables associated with HCC late recurrence.

Variables	Univariable cox regression		Multivariable cox regression	
N=133	HR(95%CI)	P value	HR(95%CI)	P value
Age, years	1.005 (0.972-1.040)	0.761		
Gender, (Female vs Male)	0.472 (0.145-1.536)	0.212		
HBsAg, (Yes vs No)	0.914 (0.401-2.081)	0.830		
Cirrhosis, (Yes vs No)	1.818 (0.834-3.961)	0.132		
Neutrophil, 10^9^/L	0.857 (0.658-1.116)	0.251		
Lymphocyte, 10^9^/L	0.574 (0.287-1.147)	0.116		
Platelet, 10^9^/L	0.994 (0.988-1.000)	0.038	0.999 (0.992-1.006)	0.760
TBil, μmol/L	1.017 (0.946-1.093)	0.655		
Alb, g/L	1.004 (0.933-1.081)	0.914		
ALT, U/L	1.004 (0.994-1.013)	0.456		
AST, U/L	0.999 (0.990 1.009)	0.859		
PT, s	1.098 (0.858-1.404)	0.458		
AFP, (>400 vs ≤ 400ng/ml)	0.936 (0.444-1.976)	0.863		
Tumor size, cm	1.016 (0.930-1.110)	0.723		
Tumor number, (≥2 vs 1)	1.575 (0.744-3.335)	0.235		
MVI, (Yes vs No)	0.889 (0.450-1.757)	0.734		
Satellitosis	2.093 (0.733-5.976)	0.168		
Splenic volume (mL)	1.003 (1.001-1.005)	0.001	1.003 (1.001-1.005)	**0.001**
BCLC staging		0.168		
A	reference			
B	1.980 (0.862-4.551)			
C	2.116 (0.634-7.059)			
Differentiation		0.237		
well	reference			
moderate	4.200 (0.574-30.734)			
poor	2.454 (0.273-22.039)			

AFP, alpha fetoprotein; HBsAg, hepatitis be antigen; ALB, albumin; TBIL, total bilirubin; ALT, alanine transaminase; AST, aspartate transaminase; PT, prothrombin time; MVI, microvascular invasion; BCLC, Barcelona Clinic Liver Cancer. ‘P < 0.05’ was highlighted in bold.

### Comparison Between Two Groups

Clinicopathological factors are compared according to two groups (shown in **Table 4**). Patients with low SV had a higher platelet count than those with high SV (189.49 ± 55.92 vs 145.06 ± 56.05, P<0.001). Also, the proportion of cirrhosis is lower in the low SV group (55.6%vs78.4%, P=0.006). However, there was no significant difference between the two groups in tumor-related factors, such as tumor size (P=0.071), AFP (P=0.436), MVI (P=0.729), BCLC staging (P=0.073), and tumor differentiation (P=0.084). Of 133 included patients, 33 out of 88 (37.5%) patients developed a recurrence in the high SV group, and six out of 45 (13.3%) patients developed a recurrence in the low SV group (P=0.004). Therefore, we can get the conclusion that after excluding tumor-related factors (related to early recurrence), splenic volume is the only predictive factor for late HCC recurrence.

**Table 4:** Comparison of clinicopathological factors between high splenic volume and low splenic volume group.

	High splenic volume (N=88)	Low splenic volume (N=45)	P value
HBsAg (+), n (%)	71 (80.7) 69 (78.4)	35 (77.8) 25 (55.6)	0.694 0.006
Cirrhosis, n (%)			
Platelet,10^3^L (mean ± SD)	145.06 ± 56.05	189.49 ± 55.92	<0.001
Tumor size, cm (mean ± SD)	5.40 ± 3.78	4.43 ± 2.30	0.071
AFP, ng/ml, n (%) ≤400 >400	68 (77.3) 20 (22.7)	32 (71.1) 13 (28.9)	0.436
Tumor number, n (%) 1 >2	71 (80.7) 17 (19.3)	38 (84.4) 7 (15.6)	0.593
MVI, n (%) No Yes	60 (68.2) 28 (31.8)	32 (71.1) 13 (28.9)	0.729
Satellitosis, n (%)	10 (11.4)	0	0.045
BCLC staging, n (%) A B C	71 (80.7) 11 (12.5) 6 (6.8)	40 (88.9) 5 (11.1) 0	0.073
Differentiation, n (%) Well Moderate Poor	6 (6.8) 73 (83.0) 9 (10.2)	4 (8.9) 30 (66.7) 11 (24.4)	0.084
Recurrence, n (%)	33 (37.5)	6 (13.3)	0.004

Data are expressed as mean ± SD or n (%).

AFP, α-fetoprotein level; HBsAg, hepatitis be antigen; MVI, microvascular invasion; BCLC, Barcelona Clinic Liver Cancer.

### Nomogram for Late Recurrence Based on SV

**Figure 6:**
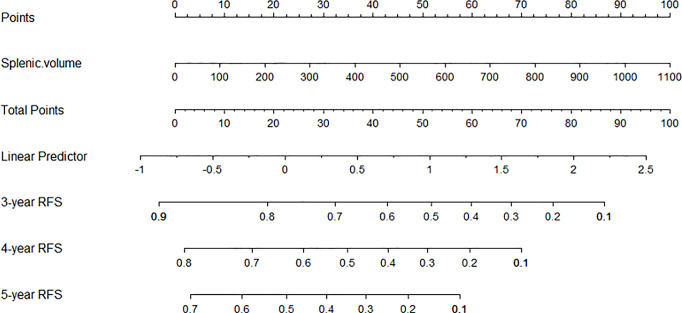
The nomogram was developed based on SV. An individual SV value is drawn upward to determine points. Because there is only one variable, the points are the total points, and a line is drawn downward to the likelihood of 3-year RFS, 4-year RFS, and 5-year RFS.

Nomograms are novel prediction models widely used for cancer prognoses such as recurrence or death (21). A late recurrence prediction model of HCC patients was conducted based on splenic volume. By calculating the preoperative SV of patients, we could simply get the probability of 3-year RFS, 4-year RFS, and 5-year RFS (shown in **Figure 6**).

## Discussion

Although surgical resection is beneficial to HCC patients, the high recurrence rate of HCC patients after hepatectomy remains the most serious challenge for both clinical surgeons and patients (9). Therefore, to better improve the management of these patients, identifying predictors of recurrence after hepatectomy is indispensable.

The determination of cut-off time between the early and the late recurrence has always been a focus of debate because of their distinct physiopathologic mechanism and strategies of surveillance, prevention, and management of HCC recurrence. Although a few studies (22, 23) suggested that less than 1 year from resection to recurrence has a worse prognosis, the cut-off of 2 years after hepatectomy has been widely accepted by researchers (6–9). Our study that there are completely different independent factors between early and late recurrence may indirectly support this point of view.

The risk factors for early HCC recurrence are mainly tumor-related, which is consistent with our study. In our study, we found that AFP (>400ng/ml), MVI, satellitosis, and BCLC staging were independent risk factors of HCC early recurrence through the multivariable Cox regression analysis. AFP serves as a surrogate marker for tumor differentiation as well as vascular invasion and high-level AFP was reported to be linked to HCC recurrence (24, 25). Microvascular invasion (MVI) is defined as the presence of tumor cell clusters in the branch of the portal or hepatic vein under microscopy (26). Obviously, the presence of MVI signifies the invasion of tumor cells into the vessel. Roayaie et al. suggested that MVI is an extremely important factor that can accurately predict the risk of recurrence (27). As for satellitosis, it refers to small metastases around the main tumor. In general, satellitosis originated from MVI, indicating that tumor metastasis has entered a late pathological stage (28). Several articles (29, 30) revealed that HCC patients with satellitosis had a worse overall survival than those without satellitosis. Our study further confirmed that satellitosis is a risk factor for early recurrence. Among many liver cancer staging systems, The Barcelona Clinic Liver Cancer (BCLC) system is currently the most widely applied system around the world. The unique advantage of BCLC staging is that the patient’s performance status, tumor burden, and liver function are considered comprehensively (31, 32). Advanced-stage HCC (BCLC-C HCC) patients have tumors that have spread beyond the liver, as well as vascular invasion. Similarly, BCLC staging has been widely validated and associated with the prognosis of HCC patients by several multicenter studies (33, 34).

Subsequently, patients without early recurrence were accepted into our further analysis for late recurrence. After multivariate analysis, splenic volume was the only predictor of HCC late recurrence. The curves for late HCC recurrence-free survival showed a statistically significant difference: patients with SV ≥165 ml had a higher recurrence rate. And for patients with SV <165 ml, fewer than half of them relapsed during the study period. Through comparison between the low SV and the high SV groups, we found that most tumor-related variables (tumor size, AFP, MVI, BCLC staging, and tumor differentiation) didn’t add a contribution to late recurrence. So according to the cut-off of 165 ml, we could preoperatively classify patients as high or low late recurrence of the crowd based on actual splenic volume (after excluding early recurrence-related factors). To obtain the late recurrence rate more accurately, we developed the nomogram based on SV. Depending on the specific value of SV, we could get the likelihood of the 3-year RFS, 4-year RFS, and 5-year RFS, which greatly facilitates individualized clinical surveillance and prevention of HCC recurrence.

There is no doubt that the risk factors for early HCC recurrence are biological characteristics of the tumor (10–12). Whereas, there are poor data for predicting late recurrence (35). It is widely acknowledged that the severity of chronic liver conditions (like liver cirrhosis) plays a significant role to some degree (13, 14). It is proven that the severity of portal hypertension accelerates the natural process of chronic liver disease, including HCC occurrence (36). Admittedly, the recurrence of HCC after 2 years of surgery was considered as a result of the development of *de novo* tumors or new malignant clones (9), which additionally confirms that late recurrence is correlated to the underlying liver condition. Therefore, we could assess the degree of liver cirrhosis by calculating the level of portal hypertension. In clinical, the Hepatic Venous Pressure Gradient (HVPG) is viewed as the gold standard for diagnosing PH. Due to its invasion, many scholars were devoted to searching for a non-invasive and simple method to evaluate HVPG. Both spleen size and platelet count were reported to serve as surrogate markers in assessing HVPG (37). In our univariate analysis for late recurrence, splenic volume (P=0.001) and platelet count (P=0.038) are risk factors, which supported previous theoretical research. It is well known that thrombocytopenia is caused by hypersplenism secondary to splenomegaly and is also one of the complications of portal hypertension. In our further multivariate analysis, however, the splenic volume (P=0.001) may play a more dominant role than platelet count (P=0.760).

HCC patients are often accompanied by congestive splenomegaly due to the background of cirrhosis. It is often seen in the increase of splenic vein pressure caused by the increase of portal vein pressure, which makes it difficult for the blood of the spleen to return, and then causes the blood stasis of the spleen, resulting in splenomegaly (38). In other words, Splenomegaly is regarded as an important symbol of cirrhosis and portal hypertension. Takeishi et al. showed that splenic volume could help predict HCC prognosis after hepatectomy, and combined splenectomy with hepatectomy can reduce tumor recurrence in patients with splenomegaly (39). Some studies (40, 41) have also found that HCC patients with splenomegaly tend to have a poor liver function. And for those patients, the incidence of tumor-related complications was significantly increased after surgeries, leading to a poor prognosis. It has also been discovered that the spleen plays an essential role in immunity. In Hashimoto’s article (42), patients with splenomegaly have a higher number of CD4+ regulatory T cells, PD-L1, and PD-L2 expression cells than those without splenomegaly, implying that patients with splenomegaly have poor tumor immunity. The inhibitory influence of CD4+ Treg cells on immune function is mainly reflected in the inhibition of activation and proliferation of helper T cells and cytotoxic T cells, and it has been found that CD4+ Treg cells are involved in the formation of tumor immune tolerance and the inhibition of anti-tumor immune response (43). Similarly, cells expressing both PD-L1 and PD-L2 are crucial in the mechanism of tumor immune escape of the tumor microenvironment. In conclusion, decreased tumor immunity caused by splenomegaly may be one of the reasons for liver cancer recurrence.

For patients with large SV, liver transplantation has incomparable advantages compared with other treatment methods. It is the only method that can completely cure cirrhosis, HCC, and portal hypertension. However, because liver transplantation has high requirements for doctors, hospital equipment and conditions, as well as patient economy, not all patients have conditions to accept liver transplantation first. For those patients who cannot accept liver transplantation, the treatment of hepatocellular carcinoma should be given priority (re-resection). Therefore, for those patients with large SV, if they cannot accept primary liver transplantation (PLT), we’ll consider hepatectomy first. Salvage liver transplantation (SLT) could be performed after several times of HCC recurrence or liver failure. It was shown that overall survival and disease-free survival were the same after PLT or SLT (44).

Our study makes use of splenic volume to predict HCC late recurrence. Patients only need a preoperative CT scan, and SV is automatically obtained by using three-dimensional CT volumetry software. Another advantage of using SV as a predictor is that in comparison to liver volume, splenic volume in adults is not affected by age, BMI, or other factors (45).

However, the current study has several limitations. Firstly, the possibility of selection bias certainly exists because of its retrospective nature. All data come from a single center and the late recurrence group had a small number of patients, which might make our results less convincing. Secondly, for developing the nomogram, the perfect design is to set up a training cohort and use other data to set up a validation cohort. This is really the shortcoming of our experiment. In fact, building a nomogram is not the main point of our article. Our team did not spend a lot of words describing the nomogram. Also, three-dimensional reconstruction of the spleen is not common in other hospitals. We have difficulty getting patient data from other hospitals as a validation cohort. Thirdly, we ignored the effect of anti-tumor therapy, such as antiviral drugs and neo/adjuvant systemic therapy, on the recurrence of liver cancer after surgery because we couldn’t control the duration time and dose of therapy used by patients. A future multicenter prospective study is necessary.

## Conclusion

In conclusion, our study verified that tumor-related factors are predictors of HCC early recurrence and splenic volume was related to HCC late recurrence because of its association with cirrhosis and PH. With the increase of SV, the probability of late recurrence also increases. This finding could assist us in guiding the clinical surveillance and prevention of HCC recurrence.

## Data Availability Statement

The original contributions presented in the study are included in the article/**Supplementary Material**. Further inquiries can be directed to the corresponding author.

## Ethics Statement

The study was approved by the ethics committee of Xiangya Hospital of Central South University.

## Author Contributions

TF and LZ contributed to conception and design of the study. TF and LM organized the database. TF and GL performed the statistical analysis. TF wrote the first draft of the manuscript. TF wrote sections of the manuscript. All authors contributed to the article and approved the submitted version.

## Funding

This work was supported by the grants National Nature Science Foundation of China (NO. 81771932).

## Conflict of Interest

The authors declare that the research was conducted in the absence of any commercial or financial relationships that could be construed as a potential conflict of interest.

## Publisher’s Note

All claims expressed in this article are solely those of the authors and do not necessarily represent those of their affiliated organizations, or those of the publisher, the editors and the reviewers. Any product that may be evaluated in this article, or claim that may be made by its manufacturer, is not guaranteed or endorsed by the publisher.
