# Supplementary figures and images for: Splenic Volume, an Easy-To-Use Predictor of HCC Late Recurrence for HCC Patients After Hepatectomy
Source: Front Oncol. 2022 May 24;12:876668. doi: 10.3389/fonc.2022.876668 (PMC9172205; doi:10.3389/fonc.2022.876668)

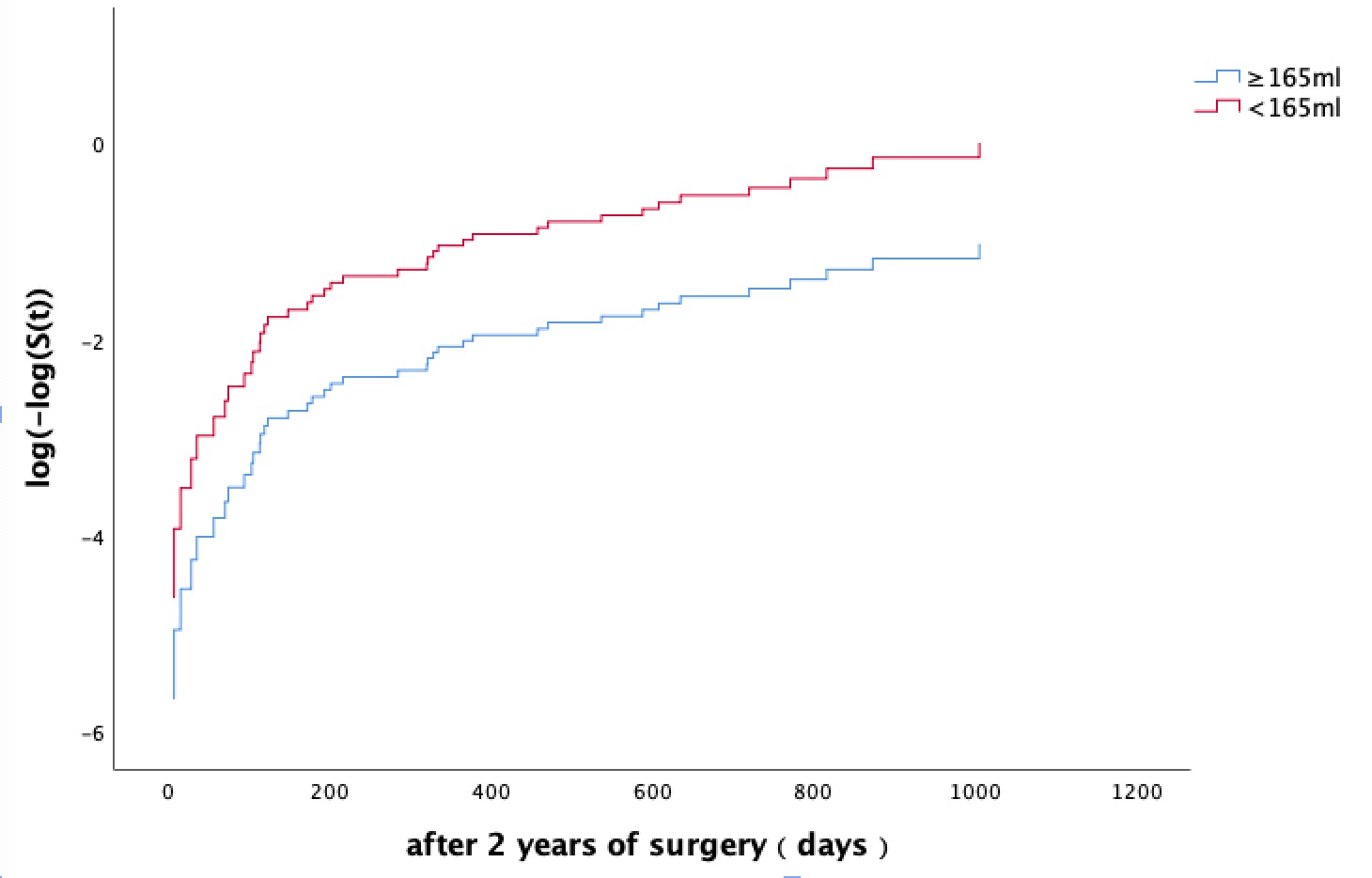

Supplement: Supplementary file 1 [file Image_1.jpeg]
